# Physical activity and quality of life in community dwelling older adults

**DOI:** 10.1186/1477-7525-7-10

**Published:** 2009-02-06

**Authors:** Siobhan M White, Thomas R Wójcicki, Edward McAuley

**Affiliations:** 1 University of Illinois, 906 S. Goodwin Ave, Urbana, IL 61801, USA

## Abstract

**Background:**

Physical activity has been consistently associated with enhanced quality of life (QOL) in older adults. However, the nature of this relationship is not fully understood. In this study of community dwelling older adults, we examined the proposition that physical activity influences global QOL through self-efficacy and health-status.

**Methods:**

Participants (N = 321, *M *age = 63.8) completed measures of physical activity, self-efficacy, global QOL, physical self worth, and disability limitations. Data were analyzed using covariance modeling to test the fit of the hypothesized model.

**Results:**

Analyses indicated direct effects of a latent physical activity variable on self-efficacy but not disability limitations or physical self-worth; direct effects of self-efficacy on disability limitations and physical self worth but not QOL; and direct effects of disability limitations and physical self-worth on QOL.

**Conclusion:**

Our findings support the role of self-efficacy in the relationship between physical activity and QOL as well as an expanded QOL model including both health status indicators and global QOL. These findings further suggest future PA promotion programs should include strategies to enhance self-efficacy, a modifiable factor for improving QOL in this population.

## Introduction

The demographic landscape of the United States is changing rapidly, with older adults representing the fastest growing segment of the population [1]. It has been well-established that the aging process can be associated with increased susceptibility to chronic conditions, disability, and comorbidity, which often results in reductions in quality of life (QOL). Physical activity has been consistently associated with enhanced QOL [2-4]; however, few efforts have been made to determine whether this relationship is direct or whether it potentially operates through other psychosocial factors.

The traditional approach in the physical activity literature has been to conceptualize QOL as representing physical, mental, and social indicators of health status, or health-related quality of life (HRQL; [5]). Stewart and King [5] adopted this approach to explain the relationship between physical activity and QOL in older adults by conceptualizing QOL as an overarching term with other factors, such as function and well-being, influencing the effect of physical activity on QOL. More recently, McAuley and colleagues [2] have tested several alternative models of the physical activity and QOL relationship in a sample of older women. In these models, they adopted Diener and colleagues' [6] position that QOL is a global construct reflecting a cognitive judgment of an individual's life. This contrasts with more traditional approaches to HRQL which view physical and mental health status as QOL outcomes. McAuley et al. [2] argued that HRQL represents a more proximal QOL indicator than global QOL. The model that best fit their data was based on social cognitive theory [7] and suggested that physical activity had a direct influence on self-efficacy [7] and, in turn, indirectly influenced QOL through indicators of physical and mental health status. Some support for such a model has also been reported in a study of individuals with multiple sclerosis [8].

In the context of older adults, a number of physical and psychosocial factors might represent mental and physical health status outcomes. For example, Elavsky and colleagues [9] have noted that self-esteem has consistently been shown to be influenced by physical activity, especially when measured from a multidimensional and hierarchical perspective [10-12]. Moreover, self-esteem has repeatedly been shown to be a strong predictor of QOL [13,14]. Importantly, self-efficacy has also been suggested to mediate physical activity effects on self-esteem [11] and some evidence exists to support this proposition [15]. Thus, self-esteem, and in particular physical self-esteem, would appear to be an important mental health status indicator in the context of the physical activity and QOL relationship. From a physical health status perspective, the likelihood of developing some type of disability increases exponentially as we age, [16] and there is evidence to suggest that disability is an important outcome of physical inactivity [17,18]. Additionally, physical activity has been suggested to offer a protective effect against functional limitations [19], a precursor to disability. Whether factors such as physical self-esteem and disabilities are implicated in the physical activity and QOL relationship, however, has yet to be determined.

Prohaska et al. [20] have made the important observation that many theoretical approaches to understanding physical activity and its consequences in older adults rarely take into consideration the role played by the demographic characteristics of participants. This may be an important issue to consider given that the lowest levels of physical activity participation are reported by adults of poorer socioeconomic status (SES) [21] and that fewer exercise facilities are found in low SES neighborhoods [22]. Furthermore, minorities typically report greater levels of sedentary behavior than their white counterparts [23]. Moreover, age is inversely related to physical activity with only 26% of individuals aged 65–74 years, and only 10% percent of those aged 85 years and over, meeting public health recommendations [24]. It is therefore important to determine whether the proposed relationships among physical activity, self-efficacy, and indicators of QOL hold when controlling for demographic influences.

**Figure 1:**
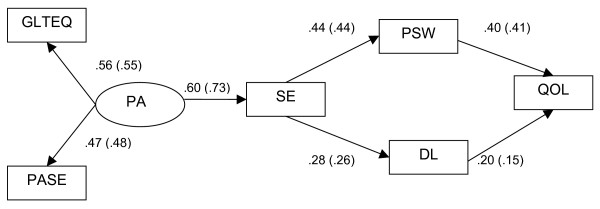
**Model of relationships between physical activity, self-efficacy, physical self-worth, disability limitations, and quality of life**. Values in parentheses represent relationships after controlling for age, income, race, education, and chronic health conditions. PA = physical activity; GLTEQ = Godin Leisure Time Exercise Questionnaire; PASE = Physical Activity Scale for the Elderly; SE = self-efficacy; PSW = physical self-worth; DL = disability limitations; QOL = quality of life.

In this study, we attempted to replicate the McAuley et al. [2] model of the physical activity and QOL relationship in a sample of community dwelling older men and women. We hypothesized that physical activity would directly influence self-efficacy, which would be associated with health status indicators. In turn, we expected health status to be associated with global QOL (see Figure 1). Finally, we examined whether these relationships were independent of the influence of demographic factors.

## Method

### Participant recruitment

We recruited community dwelling adults aged 50 and older via flyers and electronic newsletters advertising participation in a study of physical activity beliefs. A total of 349 individuals expressed initial interest and 343 individuals agreed to participate following telephone contact. We mailed a battery of questionnaires to the participants, of which 320 (93%) were returned. Incorrect or missing contact information was the primary reasons for non-participation following initial recruitment into the study.

### Measures

#### Demographics

A brief questionnaire was used to collect the demographic variables of sex, age, education, income, and race/ethnicity.

#### Physical activity

We used two self-report measures to assess physical activity participation. The first was the Godin Leisure Time Exercise Questionnaire (GLTEQ; [25]), a simple, self-report instrument assessing usual physical activity during the past seven days. This measure includes three open-ended items that measure the frequency of strenuous (e.g., jogging), moderate (e.g., fast walking), and mild (e.g., easy walking) exercises for periods of more than 15 minutes. We also measured physical activity with the Physical Activity Scale for the Elderly (PASE; [26]). The PASE is a 10-item instrument designed to assess physical activity in large samples of older persons over a one-week time period. The PASE assesses frequency and duration of participation in leisure activities (e.g., walking outside the home, light, moderate and strenuous sport and recreation) along with participation in housework, lawn work/yard care, home repair, outdoor gardening and caring for others. Scores from the PASE have been reported to be a valid measure of physical activity participation in the elderly [27,28] and are expressed as activity counts. In subsequent analyses, we modeled these two measures as a latent physical activity variable.

#### Self-efficacy

We measured self-efficacy with a modification of the Exercise Self-Efficacy Scale [29] which assesses participants' beliefs in their ability to continue exercising five times per week, at moderate intensities, for 30 or more minutes per session, and at two-week increments over the next 12 weeks. This measure has been frequently used to assess self-efficacy for physical activity [30,31] and is composed of six items scored on a 100-point percentage scale ranging from 0% (not at all confident) to 100% (highly confident). Item responses are summed and divided by six resulting in a possible range of 0–100. Internal consistency for the measure was excellent (α > .90).

#### Physical Health Status

We used the eight-item disability limitations subscale of the abbreviated Late Life Function and Disability Instrument (LL-FDI; [32]) to assess physical health status. The measure is scored on a 1 to 5 scale (1 = completely limited; 5 = not at all limited) with higher scores reflecting *fewer *limitations. This measure had good internal consistency (α = .83) and reflects physical health status in the context of carrying out household and social activities.

#### Mental Health Status

As previously noted, we characterized mental health status as self-esteem, specifically, physical self-worth, as it has been identified as a consistent psychological determinant of QOL. We used the 6-item physical self-worth scale of Fox and Corbin's Physical Self-Perception Profile [33]. A sample item from this scale is "I am extremely proud of who I am and what I can do physically." Participants indicated on a 4-point scale the degree to which each item was characteristic or true of them. Responses range from 1 (not at all true) to 4 (completely true). Internal consistency of this scale was excellent (α = .90) in the present study.

#### Quality of Life

We measured global QOL with the Satisfaction with Life Scale (SWLS; [34]), a 5-item measure, with each item rated on a 7-point scale from strongly disagree (1) to strongly agree (7). Higher scores represent greater life satisfaction. In a review of SWLS research, Pavot and Diener [35] presented evidence for the ability of SWLS to successfully detect changes in life satisfaction over time and the course of clinical interventions. The SWLS has demonstrated acceptable internal reliability and validity in older populations [35,36] and has been shown to be associated with physical activity levels [2,9]. Internal consistency in the present study was excellent (α = .90).

### Procedures

Complete details of recruitment procedures and data collection procedures can be found elsewhere [37]. Briefly, Institutional Review Board approved informed consent and all study materials were mailed to participants who then returned completed forms in a self-addressed stamped envelope whereupon participants were entered into a lottery to win one of twenty $50.00 cash prizes.

### Data analysis

We analyzed the data using covariance modeling with the full-information maximum likelihood (FIML) estimator in Mplus 5.0 [38]. In the present study, 0.9% of disability limitations data (*n *= 3), 0.3% of self-efficacy data (*n *= 1), 1.9% of GLTEQ physical activity data (*n *= 6), 1.9% of physical self-worth data (*n *= 6), 1.9% of satisfaction with life data (*n *= 6), and 6.2% of PASE physical activity data (*n *= 20) were missing.

#### Model testing

The hypothesized model proposed: direct effects of the latent physical activity variable on self-efficacy but not disability limitations or physical self-worth; direct effects of self-efficacy on disability limitations and physical self-worth but not QOL; and direct effects of disability limitations and physical self-worth on QOL. Given that the proposed model adequately fit the data, we conducted a second analysis in which the effects of demographic factors on model fit and path coefficients, as well as the model components themselves, were tested.

#### Model fit

We evaluated the fit of the proposed model for the data with the chi-square statistic, standardized root mean square residual (SRMR), and Comparative Fit Index (CFI). The chi-square statistic assesses perfect fit of the model to the data [39]. The SRMR is the average of the standardized residuals between the specified and obtained variance-covariance matrices. The SRMR should be less than .08 to indicate good model fit [40]. The CFI is an incremental fit index and tests the proportionate improvement in fit by comparing the target model to a baseline model with no correlations among observed variables. Values approximating 0.95 are indicative of good model-data fit [40]. The model tested and standardized parameter estimates are shown in Figure 1.

## Results

### Descriptive Statistics

Complete demographic details of the sample have been reported elsewhere [37]. Briefly, the sample was predominantly white (88.7%) and female (80.1%) with a mean age of 63.8 yrs (SD = 9.6). The majority of the sample (68.1%) earned $40,000 or more per year. Table 1 shows the mean scores and standard deviations for all measures included in the data analysis plus their correlations with each other. As can be seen, the sample was low to moderately active, moderately efficacious, and with few disabilities. Correlations indicated that both of the physical activity measures (i.e., PASE and GLTEQ) were significantly correlated with all model constructs with the exceptions of the association between the PASE and SWLS and the GLTEQ with disability limitations. Self-efficacy was significantly associated with all model constructs. In sum, being more active was associated with being more efficacious, having fewer disability limitations, reporting higher physical self-worth, and being more satisfied with one's life.

**Table 1:** Correlations among all model constructs

	Physical Activity Scale for the Elderly	Godin Leisure Time Physical Activity	Exercise Self-Efficacy	Disability Limitations	Physical Self-Worth	Satisfaction with Life	Mean (SD)
Physical Activity Scale for the Elderly	1.00						148.79 (80.31)
Godin Leisure Time Physical Activity	0.26**	1.00					65.89 (33.99)
Exercise Self-Efficacy	0.28**	0.33**	1.00				33.71 (34.70)
Disability Limitations	0.15*	0.08	0.28**	1.00			37.08 (4.27)
Physical Self-Worth	0.17**	0.27**	0.44**	0.23**	1.00		17.14 (4.27)
Satisfaction with Life	0.05	0.14*	0.27**	0.29**	0.45**	1.00	25.48 (6.61)

** Correlation is significant at *p *< .001

* Correlation is significant at *p *< .01

### Structural Equation Modeling of Hypothesized Relationships

The path model tested and all standardized path coefficients are shown in Figure 1. The model represented a good fit to the data, χ^2 ^= 15.59, *p *= .05; CFI = .97; SRMR = .04, meeting the accepted criteria suggested by Hu and Bentler [40] with the SRMR below .08 and CFI approximating .95. As can be seen, higher levels of the latent physical activity construct were significantly associated with greater self-efficacy (β = .60) which was, in turn, associated with fewer disability limitations (β = .28) and higher physical self-worth (β = .44). Finally, reporting fewer disability limitations (β = .20) and higher self-worth (β = .40) was associated with being more satisfied with one's life. Overall, the model accounted for 22.4% of the variance in satisfaction with life. Thus, these data would appear to support the social cognitive perspective argued by McAuley and colleagues [2] that self-efficacy and physical and mental health status variables play intermediary roles in the physical activity and QOL relationship. Additionally, the findings are supportive of the position that self-esteem, in the present context reflected by physical self-worth, is an important component of the physical activity and QOL relationship.

### Physical Activity, Quality of Life, and Demographics

As noted earlier, relationships among physical activity and quality of life have been examined relatively independent of demographic characteristics. Thus, the next model that we tested controlled for the contribution of age, race, sex, education, and income to model constructs. This allowed us to determine: (a) whether demographic characteristics changed the nature of the model relationships and (b) how demographic factors were related to individual components of the model.

This model fit the data reasonably well, χ^2 ^(13) = 38.16, *p *< .001; CFI = .93; SRMR = .04. The path coefficients of the hypothesized model were not dramatically changed, although the relationship between physical activity and self-efficacy increased from β = .60 to β = .73. All path coefficients for this model are shown in parentheses in Figure 1. In terms of the relationships among model constructs and the demographic factors, several interesting relationships emerged. Participant age was significantly (*p *<*.05*) associated with physical activity (β = -.34), self-efficacy (β = .30), physical self-worth (β = .22), and satisfaction with life (β = .12). There were less consistent patterns of significant associations among the other demographic factors and model constructs: females reported fewer disability limitations (β = -.12), white participants had a better sense of physical self-worth than other races (β = -.21), and those participants reporting higher levels of education also reported higher levels of satisfaction with life (β = .13). Finally, participants reporting higher income also reported fewer disability limitations (β = .20).

## Discussion

The purpose of this study was to determine whether the relationship between physical activity and QOL operates through self-efficacy and physical and mental health status pathways, as proposed by McAuley and colleagues [2], in a sample of community dwelling older men and women. The hypothesized associations were all significant, supporting the position that the relationship between physical activity and QOL can be understood as incorporating more proximal, modifiable, and temporally sensitive factors (e.g. self-efficacy), as well as more stable and global constructs (e.g. satisfaction with life). When we controlled for demographic variables the nature of these relationships did not change. The strengths of this study include the adoption of a well-established theoretical framework to understand the physical activity and QOL relationship, use of a relatively large community dwelling sample, and the application of contemporary statistical methods to examine the hypothesized associations.

In testing this model, we have restricted our assessments of mental and physical health status to physical self-esteem and disability frequency, respectively. In the case of esteem, we have done so because self-esteem has been frequently identified as a determinant of QOL. However, it has been demonstrated that the effects of physical activity interventions on global self-esteem have tended to be rather small [41]. This contrasts with physical activity effects on domain levels of self-esteem, i.e., the physical level [11]. Given that we have previously proposed a model of physical activity and QOL as one which capitalizes on factors which are modifiable and thereby likely to be influenced by physical activity interventions, the inclusion of physical self-esteem in concert with other indicators of mental health status may be warranted.

Similarly, there is an increasing literature which suggests that physical activity has a protective effect on functional limitations as we age [19,42]. Within the disability model framework [43], functional limitations precede disability. However, little is known about physical activity effects on disability in older adults, in large part because few physical activity studies have measured disability [44]. Even in the present sample, which was relatively disability-free, disability limitations were significantly associated with QOL and self-efficacy. Importantly, self-efficacy has previously been reported to be predictive of self-reported disability over a 30-month period in a large sample of older adults with osteoarthritis of the knee [45]. Further identification of other factors that might map onto physical and health status outcomes is called for in order to further understand the complex relationship between physical activity and QOL in older adults.

Self-efficacy, however, does appear to play an important role as both an outcome of physical activity and an antecedent of more distal QOL indicators. Perceptions of capabilities are modifiable by virtue of providing the appropriate sources of efficacy information from physical activity participation and interventions. This would suggest that such interventions can be effectively structured to maximize physical activity effects on those factors which may influence more global QOL. For example, it has been demonstrated in both cross-sectional and longitudinal designs [15,46] that self-efficacy is associated with elements of physical self-esteem reflecting physical conditioning, strength, and attractive body. Designing programs that provide information about improvements in those aspects of physical activity associated with these elements of esteem (i.e., enhancing self-efficacy) are likely to further improve physical self-worth and, in turn, QOL. In a similar vein, provision of these types of efficacy enhancing experiences can lead older adults to change their views on what might be disabling conditions or perceived frequency of disability limitations [45]. Indeed, Katula, Rejeski, and Marsh [47] have recently reported that a relatively short (12-week) intervention of high velocity power training resulted in impressive gains in self-efficacy and QOL outcomes in a sample of older adults.

Although our findings offer support for a social cognitive model of physical activity and QOL, it is not without its limitations. First, we acknowledge the cross-sectional nature of the data and therefore relationships must be interpreted cautiously. Prospective studies and randomized controlled exercise trials will be needed to determine how the proposed relationships among changes in model constructs hold up across time. Additionally, our analyses, with the exception of physical activity, were all conducted using manifest or measured constructs rather than latent variables. We believe that this is a necessity in the early stages of developing complex models of these relationships. Effectively determining which factors may or may not play an important role in representing the latent elements of physical and mental health status is necessary for further understanding their roles in this relationship. McAuley et al. [2] tested their model on a sample of older women, and although we include both males and females in our sample, the numbers of males included was substantially less than females. In this regard, our sample could be considered relatively homogenous and testing the model on more diverse samples is recommended.

## Conclusion

In conclusion, our findings support the role of self-efficacy in the relationship between physical activity and QOL, as well as an expanded QOL model including both health status indicators and global QOL. Given that the life expectancy of many countries continues to increase, a more comprehensive understanding of how we can enhance quality, as well as quantity of life would appear important. Physical activity has been consistently linked to disease risk reduction [28,48] but the manner in which it influences quality of life is not as well-understood.

Our findings have a number of implications for future research and practice. From an application perspective, self-efficacy appears to play an important role in the relationship between physical activity and quality of life. As a modifiable construct, physical activity programs that target sources of efficacy information (e.g., provision of successful experience, supportive feedback, and credible role models) are thereby likely to have a greater effect on efficacy and, in turn, enhance QOL. Such positive experiences may have implications for adherence to community exercise programs. We note that we have sampled only a few of the possible variables that act as mediators between physical activity and QOL. McAuley et al. [2] has suggested that more complex models continue to be tested. In addition, it will be important in future studies to determine whether different types of physical activity interventions differentially affect model relationships.

## Competing interests

The authors declare that they have no competing interests.

## Authors' contributions

SW, TW, EM have all made substantial contributions to conception and design, acquisition of data, analysis and interpretation of data, have been involved in drafting and revising the manuscripts, and given final approval of the version to be published.
